# Association of Protein Distribution and Gene Expression Revealed by PET and Post-Mortem Quantification in the Serotonergic System of the Human Brain

**DOI:** 10.1093/cercor/bhw355

**Published:** 2016-11-30

**Authors:** A. Komorowski, G. M. James, C. Philippe, G. Gryglewski, A. Bauer, M. Hienert, M. Spies, A. Kautzky, T. Vanicek, A. Hahn, T. Traub-Weidinger, D. Winkler, W. Wadsak, M. Mitterhauser, M. Hacker, S. Kasper, R. Lanzenberger

**Affiliations:** 1 Department of Psychiatry and Pychotherapy, Division of Biological Psychiatry, Medical University of Vienna, Währinger Gürtel 18-20, 1090 Vienna, Austria; 2 Department of Biomedical Imaging and Image-guided Therapy, Division of Nuclear Medicine, Medical University of Vienna, Währinger Gürtel 18-20, 1090 Vienna, Austria; 3 Institute of Neuroscience and Medicine (INM-2), Research Centre Jülich, 52425 Jülich, Germany

**Keywords:** MAO-A, mRNA, SERT, 5-HT1A, 5-HT2A

## Abstract

Regional differences in posttranscriptional mechanisms may influence in vivo protein densities. The association of positron emission tomography (PET) imaging data from 112 healthy controls and gene expression values from the Allen Human Brain Atlas, based on post-mortem brains, was investigated for key serotonergic proteins. PET binding values and gene expression intensities were correlated for the main inhibitory (5-HT_1A_) and excitatory (5-HT_2A_) serotonin receptor, the serotonin transporter (SERT) as well as monoamine oxidase-A (MAO-A), using Spearman's correlation coefficients (*r*_s_) in a voxel-wise and region-wise analysis. Correlations indicated a strong linear relationship between gene and protein expression for both the 5-HT_1A_ (voxel-wise *r*_s_ = 0.71; region-wise *r*_s_ = 0.93) and the 5-HT_2A_ receptor (*r*_s_ = 0.66; 0.75), but only a weak association for MAO-A (*r*_s_ = 0.26; 0.66) and no clear correlation for SERT (*r*_s_ = 0.17; 0.29). Additionally, region-wise correlations were performed using mRNA expression from the HBT, yielding comparable results (5-HT_1A_*r*_s_ = 0.82; 5-HT_2A_*r*_s_ = 0.88; MAO-A *r*_s_ = 0.50; SERT *r*_s_ = −0.01). The SERT and MAO-A appear to be regulated in a region-specific manner across the whole brain. In contrast, the serotonin-1A and -2A receptors are presumably targeted by common posttranscriptional processes similar in all brain areas suggesting the applicability of mRNA expression as surrogate parameter for density of these proteins.

## Introduction

In recent years, genome-wide transcriptome atlases have been established in rodents and more recently in humans, hereby facilitating neuroimaging research in healthy and diseased subjects (Diez-Roux et al. 2011; Hawrylycz et al. 2012; Shen et al. 2012). Kang et al. (2011) published the Human Brain Transcriptome (HBT) in 2011, including transcriptome data from 57 developing and adult post-portem brains from different brain regions. Later, the Allen Human Brain Atlas (AHBA), an extensive collection of gene expression probes across the entire adult brain, was made available for the public. However, the applicability of the atlases within the frame of neuroimaging studies is not clear yet. On a molecular level, transcripted genetic information is diversified throughout the process of protein formation as posttranscriptional, translational, and posttranslational mechanisms (PTMs) determine protein expression profiles for each cell type (Cheng et al. 2005; Kang et al. 2011). Initial mRNA transcripts interact with intra- and extracellular stimuli and are further modified, e.g. through locally regulated translation via non-protein-coding RNAs (Mattick and Makunin 2005; Di Liegro et al. 2014). Eventually, in vivo protein concentration in the central nervous system (CNS) can be quantified via positron emission tomography (PET) with radioligands that show a high specific binding for their targets and relatively low non-specific (non-displaceable) binding. Even though radiotracers frequently show considerable affinities to binding sites of other proteins, it is possible to quantify the predominant occurrence of various proteins throughout the brain in vivo (Akimova et al. 2009; Tong et al. 2013; Baldinger et al. 2014). Thereby, differences in binding affinities between agonist and antagonist radioligands exist, allowing imaging of functional conditions such as high- or low-affinity receptor states (Mongeau et al. 1992; Nenonene et al. 1994; Gozlan et al. 1995; Kumar and Mann 2014). However, availability of validated radioligands for PET imaging of the serotonergic system in humans is limited due to numerous requirements, including affinity and selectivity to the target protein.

Alterations in monoaminergic neurotransmission, which modulates cognition, mood, and emotions, as well as executive and motor functions, are seen in numerous psychiatric diseases, including subtypes of depression, anxiety disorders, and obsessive compulsive disorder (Stahl 1998; Krishnan and Nestler 2010; Hensler et al. 2013). Most notably, the serotonergic system has been within the scope of research for the last decades including molecular, genetic as well as neuroimaging studies (Gryglewski et al. 2014; Spies et al. 2015; Knudsen et al. 2016). Studies that investigated the association between mRNA and protein expression in humans, suggest an complex relationship depending on the function of genes (Celis et al. 2000; Lichtinghagen et al. 2002; Guo et al. 2008). By correlating mRNA expression from the AHBA and PET data from unrelated cohorts, Rizzo at al. showed a high association for the serotonin-1A receptor between these 2 modalities and suggested a differentiated role of PTMs for the serotonergic and opioid system (Rizzo et al. 2014). Similarly, a comparable investigation showed that monoamine oxidase-A (MAO-A) binding potentials were strongly correlated with post-mortem gene expression in healthy male controls, indicating only minor regional differences of PTMs (Zanotti-Fregonara et al. 2014).

Serotonergic cells, which are expressed in the human brain stem, innervate several cortical and subcortical neuronal systems with a broad inter- and intraregional variability. At least 14 human ionotropic and metabotropic serotonin (5-HT) receptor subtypes have been described (Nichols and Nichols 2008; Saulin et al. 2012). Also, distinct functions in cell regulation and signal transduction for several variants of 5-HT receptors (5-HT_2C5_, 5-HT_3A_, 5-HT_4_ and 5-HT_7_) as well as non-functional splicing forms (5-HT_2_, 5-HT_6_) have been reported (Xie et al. 1996; Bockaert et al. 2006; Hannon and Hoyer 2008). Regarding PET imaging, suitable radiotracers are currently only available for 5-HT_1A_, 5-HT_1B_, 5-HT_2A_, 5-HT_4_, and 5-HT_6_ receptors.

The 5-HT_1A_ receptor is distributed broadly throughout the human brain and exhibits a primarily inhibitory function. It is expressed both as a somatodendritic autoreceptor with serotonergic control mechanisms on presynaptic neurons in the dorsal and median raphe nuclei as well as a heteroreceptor on postsynaptic neurons mostly in limbic regions, including the cortex, hippocampus, septum, and hypothalamus (Sanabria-Bohorquez et al. 2002; Santana et al. 2004). Barring issues considering optimal quantification methods of 5-HT_1A_ binding potentials, the predominant consensus suggests increased binding across several brain regions in depressed patients (Kaufman et al. 2016). In contrast, 5-HT_2A_ receptors, located postsynaptically, are mainly excitatory receptors that modulate GABAergic and glutamatergic neurons (Santana et al. 2004; de Almeida and Mengod 2007). Alterations to serotonin transporter (SERT) density have been linked to genetic and hormonal influences (Kranz et al. 2015; Kraus et al. 2014). For example, low-expressing variants of the promoter region (5-HTTLPR) are associated with anxiety-like personality traits, mood disorders, increased stress sensitivity, and an altered response to selective serotonin re-uptake inhibitor treatment (Caspi et al. 2003; Clarke et al. 2010; Karg et al. 2011). Moreover, changes of the level of MAO-A and B enzymes, which are essential for maintaining a stable concentration of monoamines in the CNS, have been shown to play a role in psychiatric diseases. Especially an overactive MAO-A and associated genetic variants have been linked to brain toxicity, impulsive personality as well as to the occurrence of depressive symptoms (Du et al. 2002; Meyer et al. 2006; Kinnally et al. 2009; Lung et al. 2011; Schwartz 2013).

Given the potential of large transtriptome databases and the limited number of suitable radiotracers for PET imaging, post-mortem mRNA expression might approximate subsequent in vivo protein expression in humans as a surrogate parameter for protein density. Although interpretation is complicated due to the fact that neurotransmitter receptors and transporters are predominantly expressed either pre- or postsynaptically, while mRNA occurs mainly in the cytoplasm, effects of differing molecular localizations seem negligible considering previous studies showing strong correlations. Such studies, which compare gene expression and imaging data, have previously been performed for the serotonin-1A receptor as well as the enzyme MAO-A. In this study, we focused on the serotonergic system and addressed the role of regional differences in transcriptional processes on protein expression. Besides the main inhibitory (5-HT_1A_) and excitatory (5-HT_2A_) serotonin receptors as well as the SERT we included MAO-A, one of the prevailing enzymes responsible for degradation of monoamines. In contrast to previous correlations of mRNA expression with PET data, we not only performed a region-wise comparison, which potentially compensates effects of different molecular localizations of proteins (Rizzo et al. 2014; Zanotti-Fregonara et al. 2014), but also used a voxel-wise approach for 4 key serotonergic proteins. Applying antagonist radioligands rather than agonists we aimed to label all available receptors, regardless of their functional state for a more precise correlation with gene expression data.

## Materials and Methods

### PET Data

Cross-sectional PET data of 112 healthy subjects (60 male and 52 female) were used for the analysis. PET data for each protein are derived from a different group of subjects. Data on 5-HT_1A_ (BP_ND_) and 5-HT_2A_ (BP_P_) receptor as well as SERT (BP_ND_) binding potentials were published previously. Measurement protocols, data processing, and quantification were performed as described in detail by Savli et al. (2012).

Briefly, [*carbonyl*-^11^C]WAY-100635 binding potentials were measured to characterize 5-HT_1A_ distribution in 37 subjects (18 female, mean age = 26.9, SD = 6.7). [*carbonyl*-^11^C]WAY-100635 is a 5-HT_1A_ receptor antagonist that also shows a high in vitro affinity for the dopamine D_4_ receptor (Pike et al. 1995; Farde et al. 1997; Dilly and Liegeois 2016). Details regarding radiotracer synthesis as well as acquisition and processing of imaging have been published previously (Fink et al. 2009; Spindelegger et al. 2009).

The PET ligand [^18^F]altanserin allows for quantification of 5-HT_2A_ receptors in the human brain (Lemaire et al. 1991). For this study data from 19 subjects (8 female, mean age = 28.2, SD = 5.9) were included in the analysis (Hurlemann et al. 2008).

[^11^C]DASB was used to quantify SERT (Houle et al. 2000; Ginovart et al. 2001; Meyer 2007) in 34 subjects (11 female, mean age = 31.3, SD = 9.7) according to previously described procedures (Haeusler et al. 2009; Savli et al. 2012).

For the quantification of MAO-A distribution volume (*V*_T_) the radioligand [^11^C]harmine was applied in 22 subjects (15 female, mean age = 36.7, SD = 10.8) (Philippe et al. 2015). All enrolled participants were recruited by advertisements and gave written and informed consent. Prior to inclusion, all subjects underwent a thorough medical examination and female subjects were screened for pregnancy. Included subjects were medication-free and non-smokers as well as free of psychiatric, neurological, or somatic diseases and had no history of substance or alcohol abuse. 90 min dynamic PET scans were recorded after administration of 4.6 MBq/kg body weight of radiotracer. Analyses were performed via arterial input functions and the kinetic modeling with PMOD 3.509. Voxel-wise *V*_T_ was quantified using Logan plot (Logan et al. 1990). The delay of the arterial input function was estimated with a 2-tissue compartmental model with K1/k2 coupled across 11 representative ROIs (amygdala, hippocampal, cerebellar gray matter, cingulum, frontal, insula, midbrain, occipital, parietal, striatum, temporal, thalamus) according to Ginovart et al. (2006). Regional MAO-A distribution was extracted from the resulting parametric maps.

Study procedures were approved by the local Ethics Committee at the Medical University of Vienna. Due to reported inhibition of MAO-A activity in smoking populations all subjects were also screened for tobacco use prior to PET measurements using cotinine urine tests (Fowler et al. 1996; Leroy et al. 2009).

### mRNA Data

**Figure 1.**
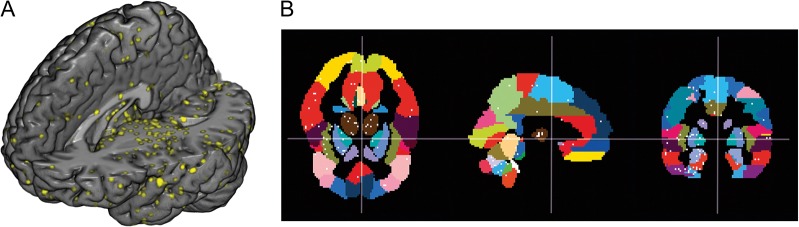
Brain topology of mRNA samples in the MNI stereotactic space. Coordinates from the AHBA are visualized by (*A*) yellow dots (8 mm³) on the MR-based brain rendering. The size of each dot exceeds the size of histological samples (0.9 mm³) for a better visualization. (*B*) White dots within color-coded AAL regions on the triplanar MNI brain template (*x* = 90, *y* = 126, *z* = 72).

Gene expression maps for all 4 proteins investigated in this study were downloaded from the freely available Allen Human Brain Atlas (www.brain-map.org). Furthermore, we downloaded mRNA values of the dopamine D_4_ receptor to test for an association with the radiotracer [*carbonyl*-^11^C]WAY-100635. The atlas offers a data set of genome-wide microarray-based gene expression profiles from up to 946 samples per brain comprising distinct anatomical structures within cortical and subcortical regions obtained from 6 healthy subjects (1 female, mean age = 42.5, SD = 13.4). Each gene included in the present analysis is represented by at least 3 probes. Details regarding mRNA quantification and data processing are reported in the supplementary data of the original publication (Hawrylycz et al. 2012; Shen et al. 2012). mRNA expression data were downloaded in log_2_-values and averaged across probes for each sample. Coordinates of all samples were extracted for subsequent voxel- as well as region-wise correlations. In the AHBA, mRNA samples are represented in Montreal Neurological Institute (MNI) space. For region of interest (ROI) analysis, mRNA samples were averaged within 54 automated anatomical labeling (AAL) regions (Tzourio-Mazoyer et al. 2002) for each brain, as well as across brains for a combined analysis. For brains in which samples were collected in both hemispheres, bilateral brain regions were averaged. For the voxel-based analysis mRNA samples from the AHBA were aligned with averaged PET data in MNI space in each donor. Additionally, all mRNA samples from 6 donor brains were added into a single brain template for the combined analysis. Overlapping samples for a voxel size of 2 mm were averaged, resulting in a total of 3428 samples (Fig. 1).

For a confirmatory analysis, mRNA expression of the 5-HT_1A_ and 5-HT_2A_ receptors, SERT as well as MAO-A from 15 age-matched adult brains (6 female, mean age = 32.0, SD = 10.1) was derived from the Human Brain Transcriptome database (http://hbatlas.org/). In total, this spatio-temporal transcriptome of the human brain includes mRNA expression values of 16 regions (cerebellar cortex, mediodorsal nucleus of the thalamus, striatum, amygdala, hippocampus as well as 11 areas of the neocortex) from 57 developing and adult brains. Initially, 1340 tissue samples from left and right hemispheres of healthy male and female donors were collected to generate the HBT database (Kang et al. 2011). Downloaded gene expression values were averaged across hemispheres as well as across donors for all anatomical brain structures provided by the HBT and subsequently matched with corresponding AAL regions to perform region-wise correlations with PET data within a single brain template.

### Statistical Analysis

Inter-subject and inter-probe correlations of imaging as well as gene expression data from the AHBA were performed by means of Spearman's correlation coefficient (*r*_s_) to ensure validity and consistency within these 2 methods. Gene expression values of each probe were correlated with values of the remaining probes for each AHBA donor brain for all proteins to calculate reliability of mRNA data. PET data of each subject were correlated with PET binding values of all other subjects at the respective mRNA sample coordinates for each radiotracer to calculate inter-subject consistency. High correlations for both data sets allowed further analysis of the correlation between PET binding potentials and mRNA expression values. Only single regional mRNA values were provided by the HBT database, therefore no inter-probe correlations were performed for HBT gene expression values.

Correlations between mRNA and averaged PET values were quantified by Spearman's correlation coefficients to account for the non-normal distribution of gene expression data. A voxel-wise as well as a region-wise correlation was performed for each protein and donor brain from the AHBA. Subsequently, both approaches were applied to a single combined data set derived from all donors. Additionally, a second region-wise analysis within a single brain template was done with HBT expression data, to compare resulting correlation coefficients.

## Results

Within the serotonergic system, consistency analyses showed strong inter-subject and inter-probe correlations for PET and mRNA derived from the AHBA, indicating feasibility of subsequent analyses for all proposed proteins (Table 1). The consistency analysis for the dopamine D_4_ receptor mRNA was rather low (data not shown). A combined region-wise correlation between [*carbonyl*-^11^C]WAY-100635 binding and gene expression values of the D_4_ receptor was weak (*r*_s_ = 0.11). Therefore, we assume that high correlations using [*carbonyl*-^11^C]WAY-100635 originate from a true association with the serotonin-1A receptor and not from a mismatch with the D_4_ receptor.

**Table 1:** Consistency analyses of protein expression within mRNA and PET data for the 5-HT_1A_ and 5-HT_2A_ receptors, SERT, and MAO-A

Protein	Mean correlation coefficients			
	mRNA	*n*	PET	*n*
5-HT_1A_ receptor	0.80 ± 0.06	3	0.88 ± 0.01	37
5-HT_2A_ receptor	0.83 ± 0.10	25	0.85 ± 0.01	19
Serotonin transporter	0.72 ± 0.16	11	0.89 ± 0.02	34
Monoamine oxidase-A	0.69 ± 0.12	31	0.86 ± 0.03	22

Mean Spearman’s correlation coefficients (*r*_s_) of post-mortem mRNA levels derived from the AHBA as well as in vivo PET binding values are reported with the corresponding standard deviation. Gene expression values were correlated between multiple probes for each histological sample of the 6 donors, while imaging data was correlated between subjects at the corresponding brain coordinates of each donor. *n* = number of probes (mRNA)/subjects (PET) for each protein.

### 5-HT_1A_ Receptor

Regarding the AHBA database, 3 probes per sample were available for the 5-HT_1A_ receptor. Inter-probe correlations of mRNA probes ranged from *r*_s_ = 0.74 to *r*_s_ = 0.89 (mean *r*_s_ = 0.80, SD = 0.06) for each of the 6 post-mortem brains. [*carbonyl*-^11^C]WAY-100635 binding potentials showed strong inter-subject consistency with correlations of *r*_s_ = 0.87–0.88 (mean *r*_s_ = 0.88, SD = 0.01) for each brain.

**Figure 2.**
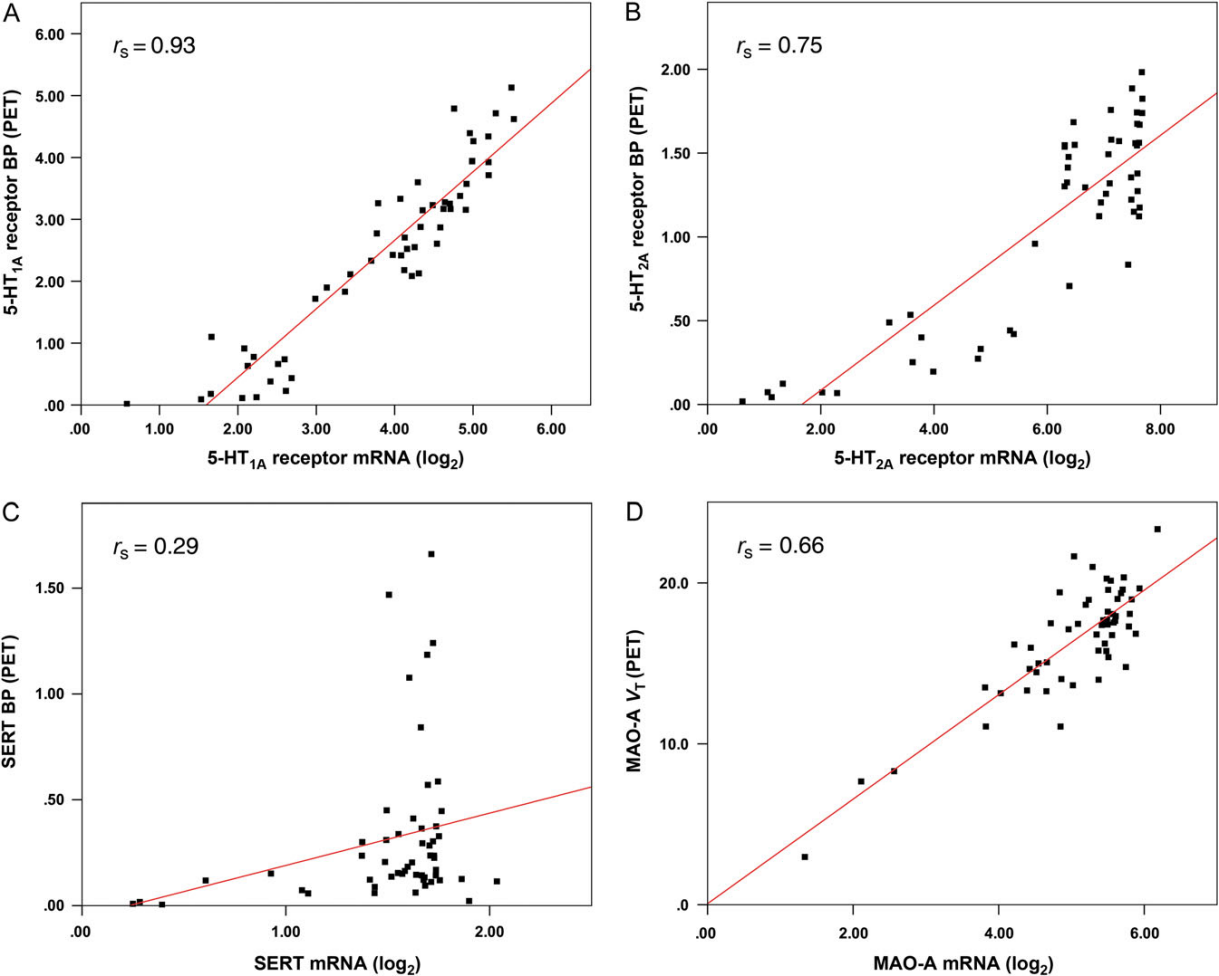
Region-wise correlations between mRNA expression (log_2_) and binding potential (BP) or distribution volume (V_T_), indices for protein concentration quantified by PET. Each dot represents averaged mRNA from the AHBA and PET values within one anatomical region. Post-mortem mRNA samples and in vivo imaging values were combined into a single brain template, for the (*A*) 5-HT_1A_ receptor (radioligand: [*carbonyl*-^11^C]WAY-100635), (*B*) 5-HT_2A_ receptor ([^18^F]altanserin), (*C*) serotonin transporter (SERT, [^11^C]DASB), and (*D*) monoamine oxidase-A (MAO-A, [^11^C]harmine).

**Figure 3.**
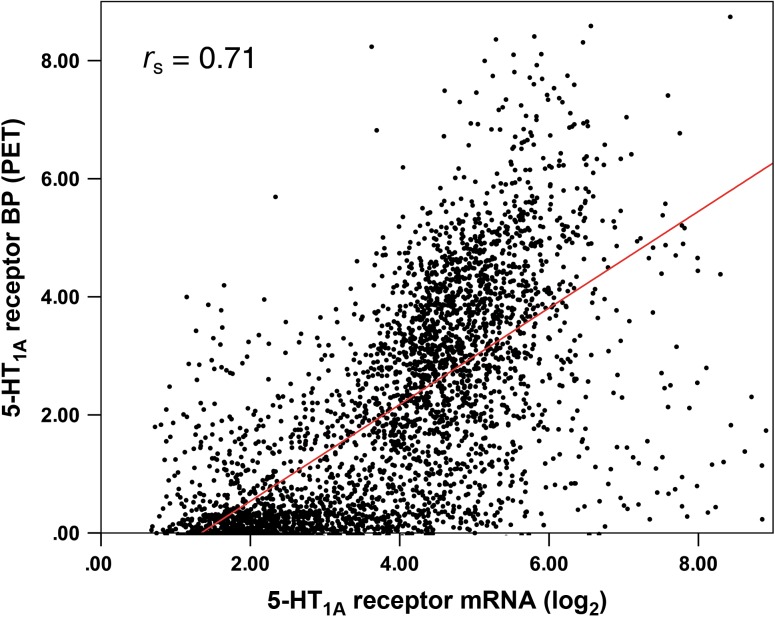
Voxel-wise correlation between mRNA expression (log_2_) and binding potential (BP_ND_) for the 5-HT_1A_ receptor (radioligand: [*carbonyl*-^11^C]WAY-100635). Each dot represents mRNA from the AHBA and PET values in target coordinates. Post-mortem samples of 6 donors and cooresponding PET binding values were combined into a single brain template.

When analyzing mRNA and PET data, consistent results were found both in the voxel-wise as well as the region-wise approach with strong associations for all brains from the AHBA. In general, correlations were higher for the ROI-based analysis in each brain (*r*_s_ = 0.71–0.92, mean *r*_s_ = 0.85, SD = 0.08) as well as for the combined correlation with inclusion of all mRNA samples (*r*_s_ = 0.93, Fig. 2*A*). In contrast, voxel-based results showed a correlation of *r*_s_ = 0.65–0.77 (mean *r*_s_ = 0.71, SD = 0.04) for single brains and *r*_s_ = 0.71 for the combined analysis (Fig. 3). The combined correlation of PET data and mRNA from the HBT within one single brain template yielded similar results (*r*_s_ = 0.82).

### 5-HT_2A_ Receptor

Similar to the 5-HT_1A_ receptor, both PET and mRNA data showed high consistency for the 5-HT_2A_ receptor. Inter-probe correlations of 25 available probes for each of the 6 brains from the AHBA showed values from *r*_s_ = 0.63 to *r*_s_ = 0.90 (mean *r*_s_ = 0.83, SD = 0.10). For [^18^F]altanserin inter-subject correlations of binding potentials ranged from *r*_s_ = 0.84 to *r*_s_ = 0.87 (mean *r*_s_ = 0.85, SD = 0.01), respectively.

PET versus mRNA correlations for the 5-HT_2A_ receptor also showed higher values for the region-wise analysis with *r*_s_ = 0.63–0.91 (single brains; mean *r*_s_ = 0.76, SD = 0.10) and *r*_s_ = 0.75 (combined samples, Fig. 2*B*), regarding the AHBA. Voxel-wise correlations yielded *r*_s_ = 0.59–0.77 (mean *r*_s_ = 0.66, SD = 0.07) and *r*_s_ = 0.66 (combined), respectively. Using the HBT mRNA database, the combined analysis showed a strong correlation for the HT_2A_ receptor as well (*r*_s_ = 0.88).

### Serotonin Transporter

Inter-probe and inter-subject correlations were between *r*_s_ = 0.43 and *r*_s_ = 0.83 (mean *r*_s_ = 0.72, SD = 0.16) for mRNA probes from the AHBA and between *r*_s_ = 0.85 and *r*_s_ = 0.90 (mean *r*_s_ = 0.89, SD = 0.02) for [^11^C]DASB, respectively. The results indicate a weaker consistency for one brain, compared to the serotonin receptors, but overall sufficient inter-probe correlations of mRNA data.

In contrast to the 5-HT_1A_ and 5-HT_2A_ receptors the SERT showed very heterogeneous ROI-based correlations for the 6 AHBA brains (*r*_s_ = 0.09–0.52; mean *r*_s_ = 0.22, SD = 0.17) and also an overall low association for the combined analysis (*r*_s_ = 0.29, Fig. 2*C*). Further, the voxel-based approach showed no meaningful correlations for SERT for each brain (*r*_s_ = −0.02–0.25; mean *r*_s_ = 0.09, SD = 0.10) or for the combined samples (*r*_s_ = 0.17). Notably, after applying the transcriptome data from the HBT project the region-wise correlation between SERT gene and protein expression did not change markedly (*r*_s_ = −0.01).

Due to the non-monotonic relation between mean SERT mRNA from the AHBA and [^11^C]DASB data on ROI-basis, we additionally conducted a Hoeffdings's *D* measure, which enables to quantify the independence of data sets also in non-monotonic relationships. With *D* = 0.02 (*P* = 0.02) the assumption of a rather weak association between gene and protein expression was confirmed.

### Monoamine Oxidase-A

Consistency analyses of MAO-A gene expression and [^11^C]harmine *V*_T_ showed values comparable to SERT, with *r*_s_ = 0.51–0.83 (mean *r*_s_ = 0.69, SD = 0.12; mRNA) and *r*_s_ = 0.81–0.89 (mean *r*_s_ = 0.86, SD = 0.03; PET), respectively.

Correlations of MAO-A distribution volumes and mRNA expression, applying the AHBA, indicated an interesting association between mRNA expression and PET binding potentials with *r*_s_ = 0.30–0.66 (mean *r*_s_ = 0.54, SD = 0.14) for each brain (combined form: *r*_s_ = 0.66) using the region-wise analysis (Fig. 2*D*). The voxel-wise correlation indicated notably weaker effects, ranging from *r*_s_ = 0.21 to *r*_s_ = 0.37 (mean *r*_s_ = 0.27, SD = 0.06) and *r*_s_ = 0.26 (combined samples). The overall higher correlations realized with the ROI-based approach are thereby in accordance with the results for the serotonin receptors and SERT, where the voxel-wise analysis showed lower values as well. Likewise, the region-wise correlation between mRNA expression and PET binding was *r*_s_ = 0.50, when applying HBT data.

### Cortical/Subcortical Analysis

For the analyzed proteins, mRNA (AHBA) as well as protein expression differed between cortical and subcortical regions. A gap of gene expression values between cortical and subcortical regions due to anatomical distribution of the proteins and available samples of the AHBA led to higher correlation coefficients when all samples throughout the brain were included in the analysis, compared to correlations limited exclusively to cortical or subcortical regions. This was true for the voxel-wise method as well as region-wise correlations (Table 2).

**Table 2:** Region- and voxel-wise correlations between PET and mRNA data from the AHBA for the 5-HT_1A_ and 5-HT_2A_ receptors, SERT, and MAO-A

	Region-wise analysis			Voxel-wise analysis		
	Cortical *n* = 38	Subcortical *n* = 6	Whole brain *n* = 54	Cortical *n* = 1488	Subcortical *n* = 895	Whole brain *n* = 3428
5-HT_1A_ receptor	0.85	0.89	0.93	0.58	0.55	0.71
5-HT_2A_ receptor	0.37	0.75	0.75	0.20	0.11	0.66
Serotonin transporter	0.16	−0.29	0.29	0.02	0.17	0.17
Monoamine oxidase-A	0.52	0.25	0.66	0.06	0.22	0.26

Spearman's correlation coefficients (*r*_s_) are reported for cortical and subcortical regions as well as for the whole brain (including data not annotated in either cortical or subcortical regions). Post-mortem mRNA samples of 6 donors were combined into a single MNI brain template and correlated at corresponding coordinates with PET binding potential/distribution volume, indices for protein density. For the region-wise correlation, data was averaged within AAL regions.*n* = number of regions (region-wise analysis)/voxels (voxel-wise analysis).

**Figure 4.**
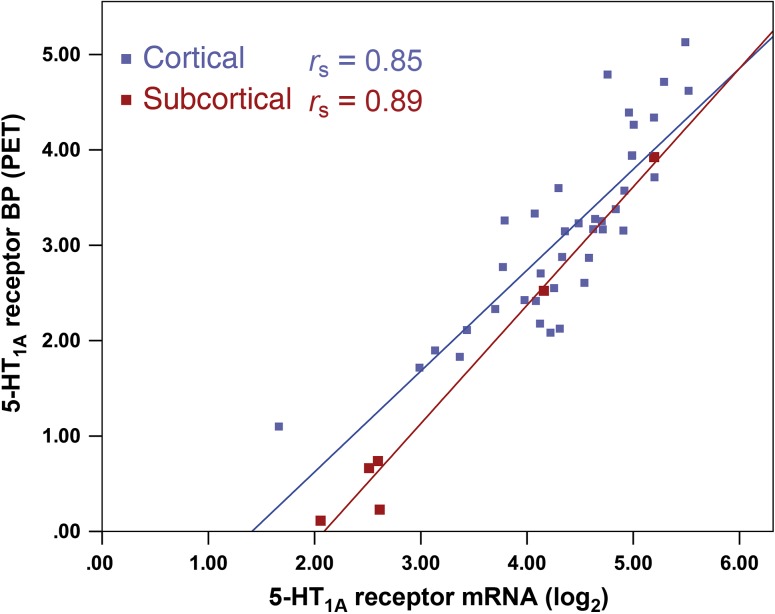
Region-wise correlation between cortical (blue) and subcortical (red) mRNA expression (log_2_) and binding potential (BP) for the 5-HT_1A_ receptor. Each dot represents averaged mRNA from the AHBA and PET values within cortical and subcortical regions. Post-mortem samples of 6 donors and corresponding PET binding values were combined into a single brain template.

For the 5-HT_1A_ receptor, strong correlations were found both for cortical (region-wise analysis, combined *r*_s_ = 0.85) as well as subcortical (*r*_s_ = 0.89) regions (Fig. 4). Nonetheless, combining all mRNA samples from the AHBA throughout all brain regions showed an overall higher correlation (*r*_s_ = 0.93). Cortical correlations (*r*_s_ = 0.37) were weak for the 5-HT_2A_ receptor, while subcortical correlations (*r*_s_ = 0.75) were as strong as correlations for the whole brain-analysis (*r*_s_ = 0.75). Regarding SERT, overall low correlations of mRNA and PET data in cortical (*r*_s_ = 0.16) and subcortical (*r*_s_ = −0.29) areas as well as in the whole brain (*r*_s_ = 0.29) showed only low or non-relevant associations. Similar to the receptors, the association between MAO-A mRNA and PET *V*_T_ was lower for the separate analyses (*r*_s_ = 0.52, cortical; *r*_s_ = 0.25, subcortical) than for the whole brain (*r*_s_ = 0.66). Regarding HBT data, we did not calculate separate correlation coefficients for cortical or subcortical regions, due to the limited number of available brain regions in this atlas.

## Discussion

**Figure 5.**
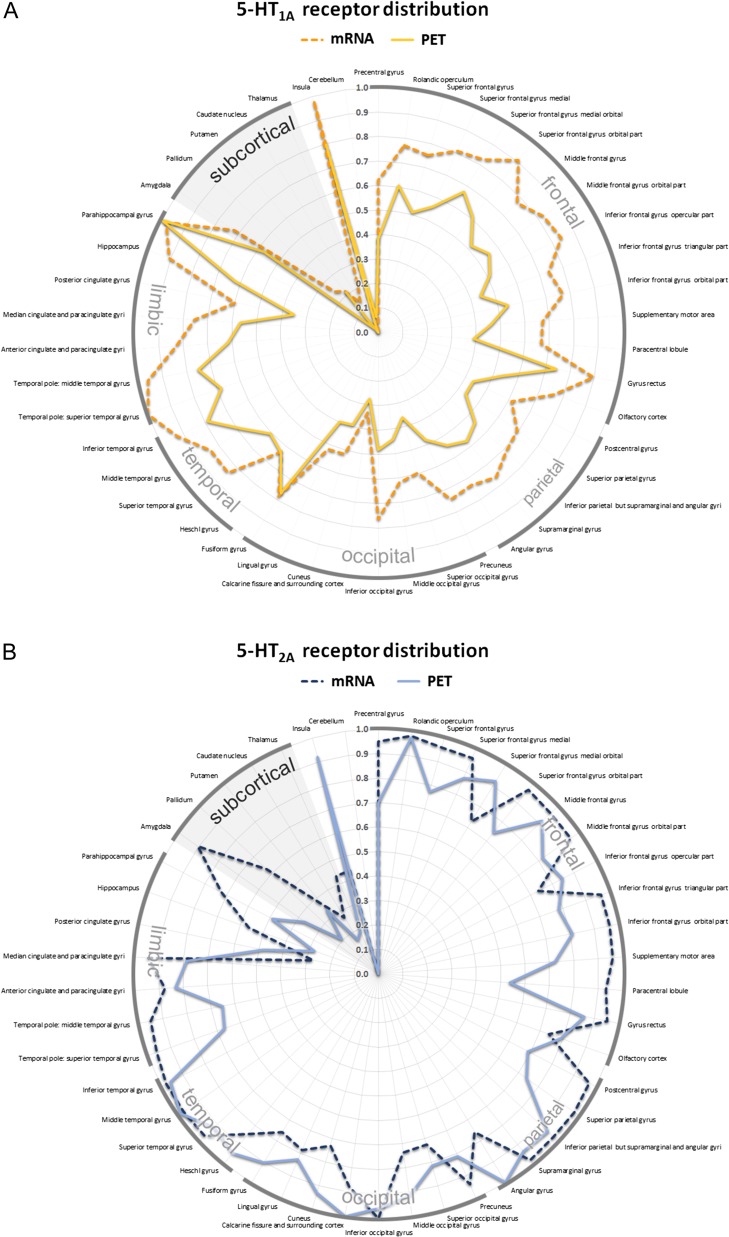

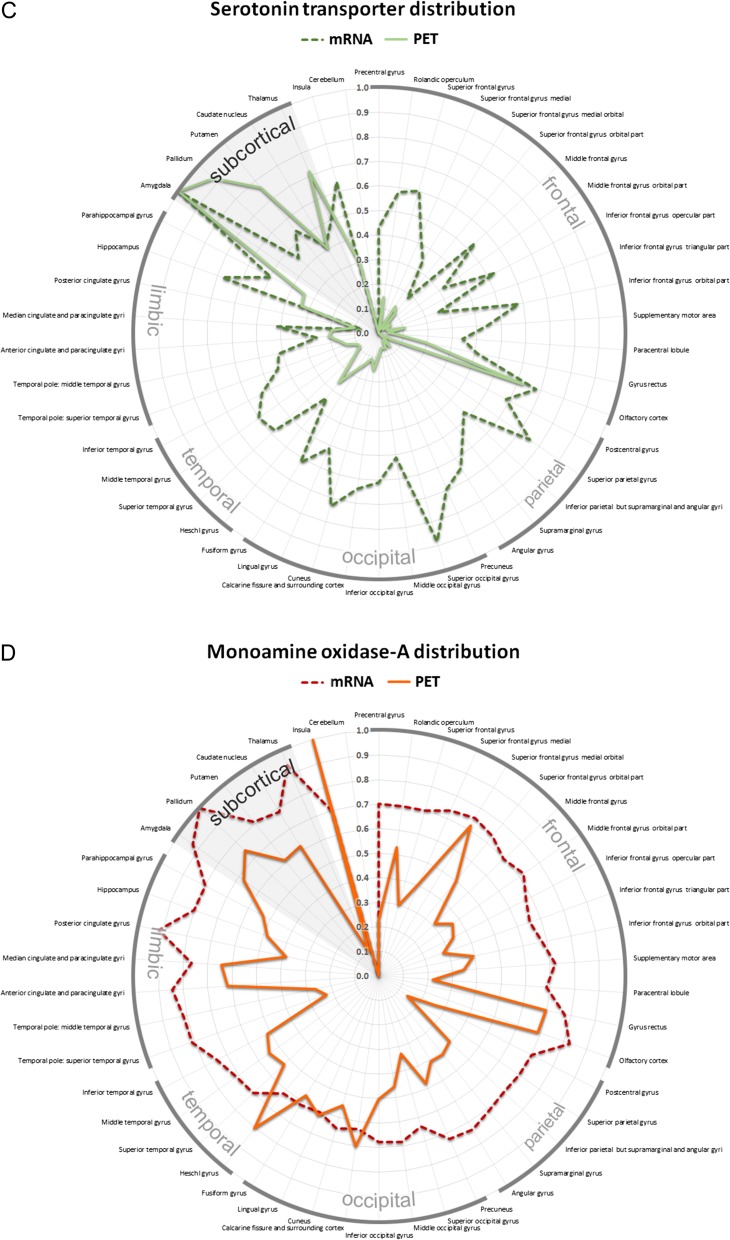
Topological distribution of serotonergic key proteins across 46 anatomical regions of interest in the human brain showed in a polar graph. The radial axis indicates PET binding (solid line) and mRNA expression derived from the AHBA (dashed line). All values were averaged for the left and right hemisphere. Absolute values of protein binding were normalized into relative values ranging from 0 to 1 for both modalities to visualize regional differences of protein distribution for the (*A*) 5-HT_1A_ (yellow) and (*B*) 5-HT_2A_ (blue) receptor, (*C*) the SERT (green) as well as (*D*) MAO-A (red).

This study provides evidence that transcriptional processes are unique and also vary between brain regions for 4 key proteins of the serotonergic system. For the investigated serotonin receptors (5-HT_1A_ and 5-HT_2A_), gene expression data from the AHBA and HBT correlates strongly with PET binding potentials, hereby indicating a strong influence of gene transcription on in vivo protein density throughout the brain for these receptors. Regardless of the applied transcriptome atlas, correlations for MAO-A are overall lower and therefore do not seem to be the result of less homogenous mRNA probes in the AHBA database. SERT shows much weaker associations of mRNA and protein binding levels, emphasizing the role of translational and PTMs, or reflecting low variability of expression between regions. Mainly postsynaptically located serotonin receptors, showed higher correlations of mRNA expression with PET binding data, compared to presynaptic proteins (SERT, MAO-A), potentially attributable to the occurrence of mRNA transcripts in the cytoplasm in the proximity of postsynaptic proteins. Certainly, results for the 5-HT_1A_ receptor need to be interpreted with regard to the simultaneous pre- and postsynaptic localization of this receptor. The relevance of studies investigating gene expression atlases arises from the limited availability of validated and accurate PET radiotracers in humans, especially for serotonin receptors and other serotonergic ligands. By utilizing the AHBA or other databases, mRNA could be applied as a surrogate parameter for protein distribution throughout the human brain, if correlated to in vivo data (Fig. 5).

In general, the cerebral cortex is represented by diverse patterns of protein expression throughout the brain dividing it into cytoarchitectural subdivisions (Zilles et al. 2002; Varnas et al. 2004). Gene transcription can be modulated through RNA binding proteins and cause changes in diseased states, most dominantly via gene silencing and alteration of mRNA metabolism (Esteller 2011; Di Liegro et al. 2014). Since densities of neurotransmitter receptors not only vary between, but also within, anatomical regions, we assumed that a voxel-wise correlation with a higher spatial resolution of mRNA values might be more specific than a region-wise analysis to examine the relationship between protein and gene expression (Eickhoff et al. 2007). By that, our primary analyses comprised all available mRNA samples derived from the AHBA localized throughout the whole brain. However, region-wise correlations with mean expression values within each anatomical region may compensate a weak co-registration as well as partial volume effects and low signal-to-noise ratio (SNR) of PET imaging in certain regions. This could explain stronger correlations for the ROI-based analyses compared to the voxel-wise approach. Although the predominant occurrence of each protein in different cell compartments might have an effect on the correlation between gene and protein expression, available transcriptome atlases do not provide an accurate mapping at a cellular resolution, thus diluting spatial allocation of mRNA samples. Gene expression values are averaged within each dissolved histological sample, including intra- as well as extracellular proteins, while PET binding shows a specific molecular topology for each radiotracer. In this respect, blocking the internalization of 5-HT_1A_ receptors in vivo has been performed, resulting in an unaltered PET signal indicating specific binding of radioligands to serotonergic receptors at the cell surface (Zimmer et al. 2004; Descarries and Riad 2012). Similarly, for [^11^C]DASB, a decrease in affinity following endocytosis of the SERT has been shown, thus implying a predominant binding of the tracer to membrane bound SERT (Quelch et al. 2012). Alternatively, [^11^C]harmine targets MAO-A, present in the intracellular compartment, which is not influenced by processes of inter- or externalization. However, associations of mRNA and PET data for extracellular proteins, the serotonin receptors and SERT, are inconsistent and indicate a rather small influence of the cellular protein localization. In a study correlating the transcriptome and the proteome in mouse brains only moderate correlation coefficients were found for each investigated cell type, also indicating rather protein-specific than cell type-related translational and PTMs (Sharma et al. 2015).

We assume similar translational and PTMs for the 5-HT_1A_ and 5-HT_2A_ receptor throughout the human brain, due to the co-expression of these proteins as well as comparable correlation coefficients found in this study (Amargos-Bosch 2004). Although it has been shown that serotonin receptors are susceptible to PTMs like phosphorylation and palmitoylation, these modifications seem to influence downstream signaling to a higher degree than cell membrane expression (Papoucheva et al. 2004; Tobin 2008). Also, no alternatively spliced functional variants of 5-HT_1A_ and 5-HT_2A_ receptors were described in psychiatric disorders so far. While serotonin receptors have been shown to activate second messenger pathways that may lead to an increase in SERT membrane density, the receptors themselves show rather stable expression patterns over time (Ahmed et al. 2008).

**Figure 6.**
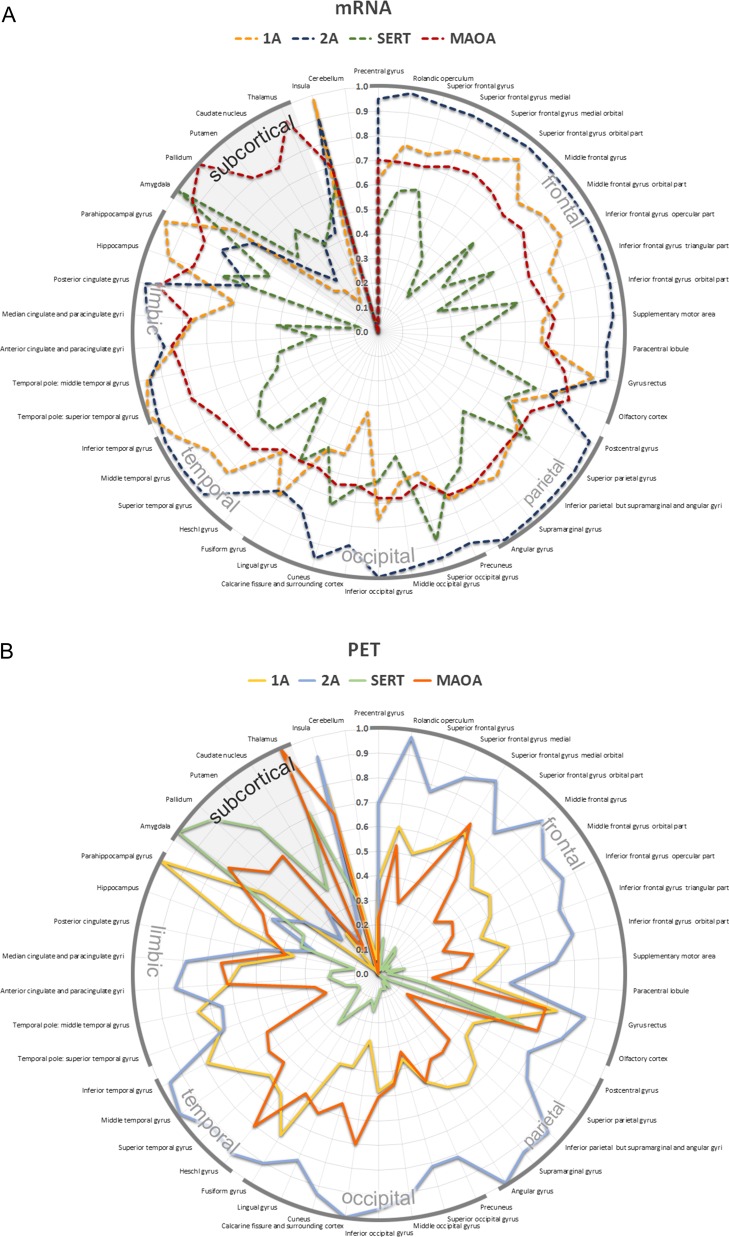
Topological distribution of the 5-HT_1A_ (yellow), 5-HT_2A_ (blue) receptor, the SERT (green) as well as MAO-A (red) across 46 anatomical regions of interest in the human brain. All values were averaged for the left and right hemisphere. Absolute values of PET binding were normalized for each radiotracer to visualize regional differences of protein distribution. For gene expression, data were normalized between proteins to visualize relative differences of mRNA expression. The radial axis indicates (*A*) mRNA expression derived from the AHBA (dashed lines) and (*B*) PET binding (solid lines).

SERT shows a presynaptic distribution, strongly depending on the presence of associated proteins as well as non-protein-coding RNAs that mediate expression levels (Austin et al. 1994; Charnay and Leger 2010). Once translated, numerous phosphorylation sites for SERT have been described to alter activity and density of the transporter, e.g. in psychiatric disorders (Miller and Hoffman 1994; Zhang et al. 2007; Steiner et al. 2009). Regarding PET imaging, SNR of used radiotracers varies clearly in different brain regions, hampering correlations across the whole brain (Meyer 2007). Results for SERT must therefore be interpreted with caution, since the SERT is found primarily in subcortical brain regions and only occurs to a lesser extent in the human cortex, which results in a lower cortical SNR for [^11^C]DASB. However, in this study voxel- and region-wise correlations for subcortical structures were comparable to cortical structures. In this respect, tissue concentrations of serotonin have been shown to be positively correlated with varying densities of the SERT indicating a regulatory role for maintaining a stable extracellular serotonin concentration (Dewar et al. 1991; Milak et al. 2005; Steiner et al. 2008). Changes of [^11^C]DASB binding potentials after pharmacological blocking of the serotonin receptor as well as changes of [^123^I]β-CIT uptake in smokers indicate a region-specific regulation for SERT (Staley et al. 2001; Baldinger et al. 2014). Further, varying SERT levels throughout the year have been reported, with higher binding potentials in fall and winter compared to spring and summer (Praschak-Rieder et al. 2008). All in all, low correlations between mRNA and PET are in line with assumed PTMs for SERT which appears to be expressed dynamically.

Notably, our results contrast previous findings demonstrating strong correlations between gene and protein expression for MAO-A (Zanotti-Fregonara et al. 2014). The region-based correlation performed previously was based on a selection of 13 ROIs. In that study, an interesting association was still present after eliminating the cerebellum as a possible confounder from the analysis. However, in vivo activity of MAO-A does not always correspond to mRNA or even protein levels, potentially due to suggested PTMs, overall reducing the validity of gene expression for this enzyme (Cao et al. 2009). Rather short-term regulatory processes are mediated via exogenous and endogenous substrates leading to an altered enzyme activity described in smokers as well as patients with depressive symptoms (Medvedev and Glover 2004; Tong et al. 2013).

Limitations to this study particularly pertain to the use of both transcriptome atlases. Available mRNA expression data does not include information about the proteome and is limited by sample quantity and quality. In the AHBA, in 4 of 6 sampled brains, gene expression is only available from one hemisphere. However, no marked interhemispheric differences of gene expression have been found in the adult brain (Hawrylycz et al. 2012; Pletikos et al. 2014). Further, several mRNA probes with low sensitivity were present in the AHBA data set, hampering overall gene expression values. Still, to assess general applicability of the atlas for serotonergic proteins, we did not select specific probes for our analysis, but chose to average mRNA expression values instead. Regarding the HBT database, gene expression values were only available in several brain regions which did not allow to perform a voxel-wise analysis. Considering different half-lives of mRNA transcripts, it can be assumed that mRNA of regulatory proteins undergoes a much faster degradation than of structural proteins, partly explaining higher correlations for these proteins in our analysis (Thapar and Denmon 2013). PET data of healthy subjects from our database were derived from unrelated cohorts, due to limitations regarding the maximum radiation doses per participant. Nonetheless, all participants enrolled in this study were selected carefully and showed similar characteristics regarding age and gender as well as the lack of psychiatric, neurological or medical diseases. Thus, even though receptor BP/*V*_T_ was calculated from 4 different cohorts the presented results collectively reflect the in vivo protein expression of healthy control subjects (Fig. 6).

The results of this study are partially consistent with previous publications showing high ROI-based correlations between genomic data obtained from the AHBA and PET imaging for the 5-HT_1A_ receptor as well as MAO-A (Rizzo et al. 2014; Zanotti-Fregonara et al. 2014). Notably, Rizzo et al. quantified binding of the 5-HT_1A_ receptor with an antagonist as well as agonist radiotracer, showing an association of mRNA with PET BP depending on the functional state of the receptor as well as the high sensitivity of [*carbonyl*-^11^C]WAY-100635 for this protein. Although an influence of dopamine receptors on the correlation using [*carbonyl*-^11^C]WAY-100635 might exist, a correlation with the D_2_ receptor was not present in that study (Rizzo et al. 2014). Likewise, we found no considerable relation of this radiotracer with the D_4_ receptor mRNA. A stronger association between gene and protein expression for cytoskeletal compared to regulatory proteins has also been shown for the 5-HT_2A_ receptor (Cornea-Hebert et al. 2002). Similarly, a varying influence of mRNA expression on protein levels depending on the function of the gene was reported in previous studies (Guo et al. 2008; Rizzo et al. 2014).

Major disadvantages of studies in neuroimaging genetics are the high costs associated with large cohorts that make collaborations between researchers increasingly necessary and data harmonization or online available databases like the AHBA or HBT even more valuable (Medland et al. 2014). While methodological advances have revolutionized biomedical knowledge about the genome and transcriptome, there is still a lack of an equally practicable atlas for the human proteome (Kim et al. 2014). Spatial matching of genomic and proteomic data may be a step towards a more comprehensive approach of the human brain, as shown in animals by Sharma et al. (2015). Further investigations, especially examining the role of PTMs in psychiatric diseases that often show a heterogeneous biological background, are necessary for a more precise understanding of the genetic context and potential changes in the serotonergic system during pathological conditions (Fried et al. 2014).

## Conclusion

These findings suggest a differentiated process of translational and posttranslational processes for the investigated proteins in different brain regions. Reflecting the high correlations for the 5-HT_1A_ and 5-HT_2A_ receptors found in this and previous studies, it might be speculated that regulation of serotonin receptor expression happens rather at the transcriptional than translational level. In contrast, we assume a potential region-specific regulation with various modifications for SERT and partly for MAO-A. A possible explanation for the differing results for pre- and postsynaptically located proteins is the role of SERT and MAO-A in the short-time regulation of serotonin levels.

By comparing 4 serotonergic key proteins, we were also able to show that mRNA data of the human brain from the AHBA may serve as an adequate surrogate parameter for in vivo protein expression of 2 serotonin receptors (5-HT_1A_, 5-HT_2A_). These results were affirmed by an additional analysis, performed with gene expression data from the HBT database. For neurotransmitter receptors lacking available radiotracers, post-mortem mRNA expression may approximate the distribution of all worthwhile proteins throughout the whole brain. Generally, our results promote a broader understanding of the serotonergic system, regarding the expression of proteins and the applicability of transcriptome atlases for further neuroimaging research. Given the different pharmacological possibilities to influence these targets further knowledge about gene expression will not only increase knowledge about psychiatric diseases but also enhance treatment strategies and advance development for adequate drugs.
